# Evolutionary diversifications of plants on the Qinghai-Tibetan Plateau

**DOI:** 10.3389/fgene.2014.00004

**Published:** 2014-02-12

**Authors:** Jun Wen, Jian-Qiang Zhang, Ze-Long Nie, Yang Zhong, Hang Sun

**Affiliations:** ^1^Department of Botany, National Museum of Natural History, MRC 166, Smithsonian InstitutionWashington, DC, USA; ^2^College of Life Sciences, Peking UniversityBeijing, China; ^3^Key Laboratory for Plant Diversity and Biogeography of East Asia, Kunming Institute of Botany, Chinese Academy of SciencesKunming, China; ^4^Institute of Biodiversity Science and Geobiology, Tibet UniversityLhasa, China; ^5^School of Life Sciences, Fudan UniversityShanghai, China

**Keywords:** evolutionary radiations, Qinghai-Tibetan Plateau, QTP, biogeography, allopatric speciation, vicariance

## Abstract

The Qinghai-Tibetan Plateau (QTP) is the highest and one of the most extensive plateaus in the world. Phylogenetic, phylogeographic, and ecological studies support plant diversifications on the QTP through multiple mechanisms such as allopatric speciation via geographic isolation, climatic oscillations and divergences, pollinator-mediated isolation, diploid hybridization and introgression, and allopolyploidy. These mechanisms have driven spectacular radiations and/or species diversifications in various groups of plants such as *Pedicularis* L., *Saussurea* DC., *Rhododendron* L., *Primula* L., *Meconopsis* Vig., *Rhodiola* L., and many lineages of gymnosperms. Nevertheless, much work is needed toward understanding the evolutionary mechanisms of plant diversifications on the QTP. Well-sampled biogeographic analyses of the QTP plants in the broad framework of the Northern Hemisphere as well as the Southern Hemisphere are still relatively few and should be encouraged in the next decade. This paper reviews recent evidence from phylogenetic and biogeographic studies in plants, in the context of rapid radiations, mechanisms of species diversifications on the QTP, and the biogeographic significance of the QTP in the broader context of both the Northern and Southern Hemisphere biogeography. Integrative multidimensional analyses of phylogeny, morphological innovations, geography, ecology, development, species interactions and diversifications, and geology are needed and should shed insights into the patterns of evolutionary assembly and radiations in this fascinating region.

## INTRODUCTION

**FIGURE 1:**
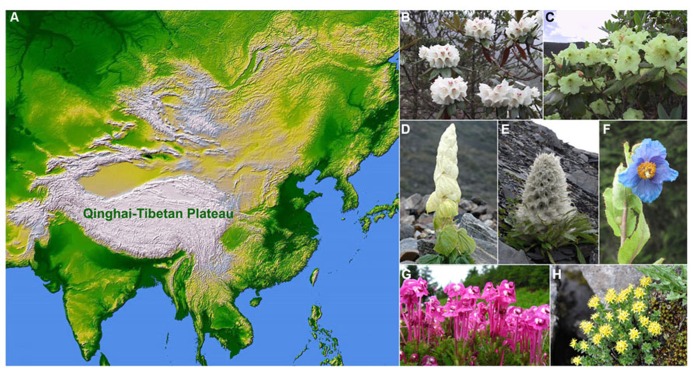
**Geographic location of the Qinghai-Tibetan Plateau (A) and representative plant groups: (B)** *Rhododendron roxieanum* Forrest ex W.W.Sm.; **(C)** *Rhododendron wardii* W.W.Sm.; **(D)** *Rheum nobile* Hook.f. & Thoms., showing “glasshouse-like” morphology; **(E)** *Saussurea laniceps* Hand.-Mazz., showing “snow-ball” morphology;****(F)** *Meconopsis betonicifolia* Franch.; **(G)** *Pedicularis siphonantha* D.Don var. *delavayi* (Franch. ex Maxim.) P.C.Tsoong; and **(H)** *Rhodiola* *alsia* (Fröd.) Fu (photo credit: **B,C,G** by H. Sun; **D,F** by J. Wen; **E** by B. Song; and **H** by J. Q. Zhang).

The Qinghai-Tibetan Plateau (QTP) is the highest and one of the most extensive plateaus in the world, covering an area of 2.5 × 10^6^ km^2^ with an average elevation of more than 4000 m (Zhang et al., 2002; Geng et al., 2009). The QTP is generally delimited by the Qilian and the Kunlun Mountains in the north, the Himalayan Mountains in the south, the Karakorum Range of Pakistan in the west, and the Hengduan Mountains in the east (**Figure 1A**; Wu et al., 1995; Turner et al., 1996; Zhang et al., 2002). The uplifts of the plateau were driven by the collision of the Indian plate with the Eurasian plate, which began at ca. 50 million years ago (mya; Coleman and Hodges, 1995; Rowley and Currie, 2006; Royden et al., 2008). Since the early Miocene, extensive uplifts of the QTP occurred in at least four major periods: 25–17, 15–13, 8–7, and 3.5–1.6 mya (see Harrison et al., 1992; Li et al., 1995; Shi et al., 1998; Guo et al., 2002; Spicer et al., 2003). The plateau harbors more than 12,000 species of vascular plants in 1500 genera, with the Hengduan–Himalayan region of the eastern and southern parts of the QTP possessing exceptional species richness and a high level of endemism (Wu, 1988; Li and Li, 1993), especially for alpine elements (Wu et al., 1995; Liu et al., 2000). Nevertheless a few recent studies examined the evolution of unique endemic plants and have found that several monotypic plant genera in the alpine regions of the QTP are nested within larger genera (see Friesen et al., 2000; Tian et al., 2011; Nie et al., 2013).

The various QTP uplift events between the early Miocene and the Quaternary, in association with the zonal climate pattern of the Paleogene controlled by desert and steppe climates (Zhang et al., 2012b) transforming into a monsoon-dominated pattern similar to the present-day one in the Neogene (Guo et al., 2008), have been shown to have triggered and facilitated plant speciation and diversifications (see Wang et al., 2007b; Qiu et al., 2011). Some of the plant lineages diverged following the early uplifts of the QTP, or even prior to the formation of the plateau (Liu et al., 2002a; Yang et al., 2003; Zhang and Fritsch, 2010; Sun et al., 2012). Most studies support divergences in the last 5 Ma, such as in *Rheum* L. (Wang et al., 2005), *Sinacalia* H. Rob. & Brettell (Liu et al., 2006), and *Solms-laubachia* (Maxim.) Botsch (Yue et al., 2009).

Many phylogeographic studies have been conducted in the past decade to explore the evolutionary consequences of climatic fluctuations and complex local topography and geomorphology of the QTP and its surrounding regions (see reviews by Geng et al., 2009; Qiu et al., 2011; Liu et al., 2012; also see Yang et al., 2009 for perspectives on animal lineages). Multiple refugia have been inferred in the peripheral regions of the QTP based on results from many plant lineages (Liu et al., 2012). The southeastern QTP has been suggested to harbor glacial refugia in several lineages of plants and animals especially during the Last Glacial Maximum (LGM; Chen et al., 2008; Yang et al., 2008a; Zhang et al., 2010; Zhou et al., 2013a). Several studies suggested refugia in the Hengduan Mountains on the eastern border of the QTP (Zhang et al., 2010; Wang et al., 2011b). It has been suggested that advances of the Tibetan glaciers were less prominent than other regions in the Northern Hemisphere, likely due to arid conditions and complex topography, and potential habitats for cold-tolerant species could be found in the plateau and served as refugia in the interior Central regions of the QTP during interglacial periods in the Quaternary (Schäfer et al., 2002; Wang et al., 2009a,2011b; Shimono et al., 2010).

A number of studies have explored macro-evolutionary relationships (Wang et al., 2005, 2009c; Liu et al., 2006; Zhang et al., 2011a; Eaton and Ree, 2013; Zhou et al., 2013b) and the impact of aridization of interior Asia on plant population structure and speciation (Zhang and Fritsch, 2010; Zhang et al., 2012a). This paper reviews recent evidence from phylogenetic and biogeographic studies in plants, in the context of rapid radiations, mechanisms of species diversifications on the QTP, and the biogeographic significance of the QTP in the broader context of Northern Hemisphere biogeography.

## SPECTACULAR RADIATIONS

Several spectacular rapid radiations (**Figures 1B–H**) have been reported to be triggered by extensive uplifts of the QTP, which caused drastic habitat fragmentation (Liu et al., 2006; Wang et al., 2007b) and resulted in the rapid expansion of cold and dry habitats by strengthening the Asian monsoon climate (An et al., 2001). The mechanisms of the QTP plant radiations and diversification certainly go beyond allopatric speciation. Other mechanisms especially pollinator-mediated isolation, hybridization and introgression, allopolyploidy, and morphological innovations have also been proposed. In a strict sense, evolutionary radiations represent many species or lineages evolved from a common ancestor in a short time period (Wen et al., 2013). Due to the fact that evolutionary radiations and species diversifications have not been well analyzed for the QTP plants, we included discussions of plant groups, which are highly species-rich on the QTP.

An extraordinary species-rich group on the QTP, especially the Hengduan Mountains is the genus *Pedicularis* L. (Orobanchaceae). The genus consists of about 600 species with over 270 species endemic to the mountains of SW China and is also widespread in alpine and cold areas of the Northern Hemisphere (Yang et al., 1998; Ree, 2005). Species of *Pedicularis* exhibit unusual diversity of floral characters (Yang et al., 2003) and they are mainly obligate outcrossers and are pollinated by bumblebees (Macior and Sood, 1991; Macior and Tang, 1997; Macior et al., 2001; Wang and Li, 2005; Eaton et al., 2012; Huang and Shi, 2013). Eaton et al. (2012) reported that local species richness of *Pedicularis* in SW China is best explained by a model including both floral diversity and phylogenetic distance. A mosaic of pollinator-mediated interactions among *Pedicularis* species promotes ecological sorting through recurrent selection against reproductive interference, which explains the rapid species turnover at local scales, and drives floral divergence among species.

Species of *Rhododendron* L. are common members of the Sino-Himalayan region in the southern part of the QTP. The genus consists of about 1025 species with many narrow endemics often sympatric with interfertile congeners throughout their ranges (Fang and Min, 1995; Milne, 2004; Goetsch et al., 2005). *Rhododendron* subgenus *Hymenanthes* appears to have undergone rapid radiations in the Sino-Himalayan region, with about 200 species in the region and the adjacent areas in China (Chamberlain, 1982; Fang and Min, 1995). Hybridizations, habitat fragmentation, and isolation through elevational changes have been hypothesized to be important for the radiation of species of subgenus *Hymenanthes *(Blume) K.Koch (Fang and Min, 1995; Zha et al., 2008; Milne et al., 2010). All *Rhododendron* subgenus *Hymenanthes *species are diploids (2*n* = 26), and hybridizations are commonly recorded among taxa, even between distantly related species (Chamberlain, 1982; Zha et al., 2008, 2010). Yet speciation via hybridization needs to be rigorously tested in *Rhododendron* on the QTP.

*Meconopsis* Vig. of Papaveraceae, the well-known blue-poppy genus of the Himalaya, consists of about 50 species on the QTP and adjacent regions in the Himalaya (Yang et al., 2012). One species, *Meconopsis cambrica* Vig. is native to Europe, and has been shown that it does not belong in the genus (Kadereit et al., 1997, 2011). *Meconopsis* is resolved as sister to sect. *Meconella* Spach of *Papaver* L. (Carolan et al., 2006; cf. Jork and Kadereit, 1995; Kadereit et al., 1997). Yang et al. (2012) suggest that geographic isolation and hybridization are two important mechanisms for speciation in *Meconopsis*, a genus also with polyploids, implying the role of allopolyploidy in the species radiation of the blue-poppy genus on the QTP.

*Rhodiola* L. (Crassulaceae) is a genus consisting of about 90 species, most of which distributed in QTP and adjacent areas (Fu and Ohba, 2001). Molecular phylogenetic studies (Zhang et al., 2014) revealed significant convergence of previously thought important morphological characters, i.e., dioecy and marcescent flowering stems, which made most of previous infrageneric taxa non-monophyletic (e.g., Ohba, 1978; Fu and Fu, 1984). The biogeographic analyses suggest a rapid radiation triggered by the uplift of the QTP during the middle Miocene (Zhang et al., 2014). Radiation continued in the later evolutionary process and some lineages dispersed into other regions (e.g., Central Asia, and North America) of the Northern Hemisphere. Allopolyploidy may also have played a role in the radiation of *Rhodiola* on the QTP, as the genus has been recorded with allopolyploids (Zhang et al., 2014).

Rapid radiations have been reported in several lineages of Asteraceae on the QTP, such as *Ligularia* Cass.–*Cremanthodium* Benth.–*Parasenecio* W.W.Sm. & J.Small (L–C–P) complex (Liu et al., 2006), the *Dolomiaea* DC.–*Diplazoptilon *Y.Ling*–Xanthopappus* C.Winkl. group (Wang et al., 2007b), *Saussurea* (Wang et al., 2009c), *Leontopodium* R.Br. ex Cass. (Blöch et al., 2010; Safer et al., 2011), and the *Soroseris *Stebbins*–Stebbinsia* Lipsch. group (Zhang et al., 2011a). In all these studies, levels of substitution and support for internal branches in the phylogenetic analyses are low, and morphologically diverse species often group together. The rapid diversification pattern has been explained by habitat fragmentation and heterogeneity followed by climatic changes during the uplift of the Himalayas and the Tibetan Plateau in the late Tertiary (Liu et al., 2006; Royden et al., 2008). We discuss the L–C–P complex of the Tussilagininae (Senecioneae) and *Sausurrea* as case studies to illustrate this common pattern of radiation on the QTP.

The L–C–P complex contains more than 200 species endemic to the QTP. The eleven genera in an expanded L–C–P complex constitute a largely unresolved L–C–P complex clade, which was suggested to have originated as a rapid radiation mostly within the last 20 Ma. The age estimates correlate with the period of recent major uplifts of the QTP between the early Miocene to the Pleistocene. Liu et al. (2006) suggested vicariance and ecological diversity associated with the QTP uplifts most likely promoted rapid and continuous allopatric speciation in small and isolated populations, and facilitated morphological convergence in the L–C–P complex. Other mechanisms including diploid hybridization in secondary sympatric conditions were proposed for the L–C–P complex as well.

*Saussurea* (Cardueae) of Asteraceae is a genus of over 450 species, with a high level of endemism on the QTP (ca. 230 species), ca. 150 species in Central Asia to the Russian Far East, and ca. 100 species in eastern Asia (Lipschitz, 1979; Kita et al., 2004; Wang et al., 2009c). The phylogenetic analyses (Wang et al., 2009c) suggest that *Saussurea* s.l. is a polyphyletic group with several parallel clades of the lineage, supporting island-like adaptive radiations in a continental setting and morphological convergences on the QTP. The dating results suggest that this radiation occurred 14–7 mya, during the period of the major uplift events of the QTP.

However, Nie et al. (2013) reported that the diversification in *Anaphalis* DC. of Asteraceae on the QTP is much more complex and cannot be explained using a single radiation event. The extant *Anaphalis* in the eastern Himalaya consists of at least four independent lineages. The diversifications of these four groups followed different patterns. Clade I contains only one species commonly found from the western to the eastern Himalaya, extending as far as southeastern Asia to the Philippines. There is apparently no radiation in clade I but its wide distribution has perhaps resulted from dispersals. The widest distribution of clade II among the four *Anaphalis* groups, ranging from central Asia through the Himalaya to northeastern and southeastern Asia, is probably due to dispersal. The intercontinental disjunction between North America and Asia within this clade was hypothesized to have resulted from a recent dispersal event from Asia to North America. However, the poorly resolved relationships of taxa within clade II may be due to a rapid radiation. Seeds in *Anaphalis* can be small and light, and possess a pappus, which may be well adapted for wind dispersal. Clade III is composed of about four species and they are mainly restricted to the Himalayan region (Ling, 1979). However, little sequence variation was observed within this clade in spite of the highly distinctive morphology between* A. busua* DC. and the *A. contorta* Hook.f. group. Clade IV is a highly diversified group in the eastern Himalayas and the Hengduan Mountains of SW China, but species relationships within this clade in general are weakly supported due to low rates of sequence divergence, perhaps supporting an evolutionary radiation.

## MECHANISMS OF SPECIES DIVERSIFICATION ON THE QTP

Below we discuss these mechanisms with a few case studies. For a given plant group, more than one such mechanism may have played a role, even though we discuss them separately.

### ALLOPATRIC DIVERGENCE

Vicariant allopatric speciation associated with the geologic uplifts has been proposed as the main mechanism of species diversification of plants and animals on the QTP (Yang et al., 2009; Xu et al., 2010). The plateau has many high mountains and plains incised deeply by numerous valleys or rivers, forming an extremely complex topography with diverse habitats.

*Phyllolobium* is a genus as a recent segregate from *Astragalus*, and contains about 20 species and four sections mostly endemic to the QTP. Zhang et al. (2012a) hypothesized *Phyllolobium* as a recently diversified genus adapted to the cold and dry habitats of the QTP. The crown of *Phyllolobium* was dated to the Pliocene: 3.62–3.96 Ma, and sections within the genus had ages ranging between 3.60 and 2.55 Ma. These dates coincide with the intense uplift of the QTP in the late Pliocene. The diversification of sections *Bibracteolati* and *Oliganthum* began at estimated ages of 1.95 and 1.83 Ma, respectively. The estimated crown ages of *Phyllolobium* and its sections suggest that rapid diversification was likely triggered by consecutive phases of the QTP uplifts in the late Pliocene and the early-to-mid Pleistocene. The diversification of some *Phyllolobium* species can be explained by late Pleistocene glaciations and/or geologic events.

Xu et al. (2010) report a case study on eight *Cupressus* species, with each having a mainly allopatric distribution on the QTP and adjacent regions. The phylogenetic and network analyses showed that most DNA haplotypes recovered or haplotype lineages resolved were species-specific, and are consistent with allopatric divergences.

Qin et al. (2013) investigated the evolutionary history of 14 *Ephedra* species from the QTP and northern China. The phylogeographic and phylogenetic analyses support three main lineages (eastern QTP, southern QTP, and northern China) of these *Ephedra* species. Divergence of each lineage was dated to the middle-to-late Miocene, a period with the active uplifts of the QTP and the Asian aridification.

*Soroseris*, *Stebbinsia*, and *Syncalathium* of Asteraceae are three genera endemic to the high screes of the QTP (Zhang et al., 2011a). The diversification of these groups in the Tibetan Plateau is of relatively young age, with the stem and crown ages of the *Soroseris*–*Stebbinsia* clade and the two groups of *Syncalathium* estimated between 8.44 and 1.56 Ma. The evolution of the groups on the QTP is hypothesized to be characterized by rapid diversification and radiation of the *Soroseris*–*Stebbinsia* clade, allopatric speciation within *Syncalathium* s. str. and convergent evolution of *Syncalathium *s.str. and *Syn. souliei*. The speciation events are correlated with climatic changes and fragmentation of scree habitats during the uplifts of the QTP.

*Cyananthus* Wall. ex Benth. (Campanulaceae) contains ca. 20 species with a typical Sino-Himalayan distribution (Hong et al., 2011). Most *Cyananthus* species are prominent components of alpine meadows at high altitudes in the Sino-Himalayan region, and many species are narrow endemics with a few widespread taxa (Hong and Ma, 1991; Shrestha, 1997). Zhou et al. (2013b) reconstructed the processes of biogeographic diversification in the region using *Cyananthus* as a model. Molecular dating and biogeographic analysis support dispersal from the Himalayas to the Hengduan Mountains during the early evolution of *Cyananthus*. The early diversification event that resulted in the split of the two main lineages within *Cyananthus* was estimated to have taken place between 5.98 and 12.46 mya, coinciding with the third extensive period of the QTP uplift since the Miocene. Zhou et al. (2013b) suggests that dispersal was the main mechanism of early diversification of *Cyananthus*, and that the vicariance triggered by the uplift of the QTP accelerated the divergence between sect. *Cyananthus* and the two other sections of the genus, with sect. *Cyananthus* mainly in Himalayas and the two other sections originated in the Hengduan regions.

At the shallower intraspecific level, vicariance by topography has been reported in a large number of recent phylogeographic analyses, such as in *Aconitum gymnandrum* Maxim. (Wang et al., 2009a), *Hippophae tibetica* Schltdl. (Jia et al., 2011), and *Incarvillea sinensis* Lam. (Chen et al., 2012), suggesting the ongoing contribution of vicariance on the QTP to plant evolution and differentiation.

### CLIMATIC OSCILLATIONS AND DIVERGENCES

The Tertiary and Quaternary climatic oscillations associated with the QTP uplifts have been widely discussed in facilitating speciation and diversification (Sun, 2002a,b), and shaping geographic genetic structure and the recolonization patterns from multiple refugia on the QTP (Cun and Wang, 2010; Qiu et al., 2011; Liu et al., 2012) and in a broader geographic context (see Hewitt, 2000, 2004). The rapid uplifts of the QTP have also led to drastic climatic divergences on the plateau, which have been largely controlled by the Indian summer monsoon (Wang and LinHo, 2002). The Indian summer monsoon has brought heavy summer rainfall to the southern part of the Himalayas, with decreasing precipitation in other areas of the QTP since the late Miocene (An et al., 2001). The impact of the climatic oscillations and divergences has been confirmed in various groups of plants (Yang et al., 2012; Zhou et al., 2013b), with many recent studies emphasizing the glacial and interglacial climatic oscillations in the Quaternary (see review by Qiu et al., 2011). The recent phylogeographic study of *Sophora davidii* Kom. ex Pavol. also provides support for a climatically driven barrier to present-day plant dispersal across the “Tanaka-Kaiyong Line” (Fan et al., 2013a).

### HYBRIDIZATION AND INTROGRESSION

Interspecific diploid hybridization has been suggested to have contributed to the high species diversity on the QTP and adjacent areas, because of secondary sympatry during relatively stable stages between different uplifts of the QTP (Wang et al., 2001; Liu et al., 2006). Hybridization has been proposed to have played an important role in the spectacular radiations of several plant groups such as *Rhododendron* at the southern part of the QTP in the Sino-Himalayan region (Zha et al., 2010; also see Milne et al., 2010), and *Meconopsis* on the QTP (Yang et al., 2012). The homoploid hybrid speciation of *Pinus densata* Masters, a high mountain pine endemic to the QTP has been rigorously documented in a series of studies with the parental species as *P. tabulaeformis* Carrière and *P. yunnanensis* Franch. (Wang et al., 2001, 2011a; Song et al., 2002, 2003; Ma et al., 2006). Nevertheless, although both hybridization and introgression have been confirmed among species in nature on the QTP (e.g., Li et al., 2008; Zhu et al., 2009), the general impact of hybridization and introgression on plant speciation on the QTP has remained poorly studied so far.

### MORPHOLOGICAL CONVERGENCE AND INNOVATIONS

A number of previous studies have shown that rapid species diversification occurred in response to the extensive uplift of the QTP and resulted in numerous morphologically distinct species, in which morphological traits appear to be ecologically adaptive, thus extensive morphological convergences have been documented (Liu et al., 2006; Wang et al., 2009a; Xie et al., 2011; Zhang et al., 2014). Species on the QTP from different lineages might have been under similar selection pressure after the uplifts of the plateau. This may explain the extensive morphological convergent evolution observed. For example, *Parasyncalathium souliei* (Franch.) J.W.Zhang, Boufford & H.Sun was once treated as a species of *Syncalathium* Lipsch. due to very similar morphological outline to other species of *Syncalathium* (Crepidinae); and it has been recently transferred into Lactucinae as a new genus based on recent molecular, cytological and morphological evidence (Zhang et al., 2011b). Other well-known examples of morphological convergences of the QTP plants include “cushion” plants (Li et al., 1985; Wang et al., 2004), “glasshouse” morphology (Ohba, 1988), and “snow-ball” plants with densely villous aggregated globose or spiciform synflorescences (Ohba, 1988; Tsukaya and Tsuge, 2001; Tsukaya et al., 2002; Kita et al., 2004; Yang et al., 2008b).

The “glasshouse-like” morphology of plants on the QTP is a well-known morphological innovation and is worthy of more discussions. The upper leaves of plants have developed into large translucent cream-colored bracts that cover the inflorescences (Tsukaya and Tsuge, 2001; Yoshida, 2002). These bracts may play a critical role in protecting pollen grains from damage by low temperatures and ultraviolet (UV) radiation (Omori et al., 2000), maintaining warmth inside the inflorescence (Terashima et al., 1993), and enhancing seed productions (Yang and Sun, 2009; Song et al., 2013). The “glasshouse” morphology has been documented in more than 10 plant families, which indicates obvious convergence of this character in diverse groups (Ohba, 1988). Phylogenetic studies including both “glasshouse” plants and “non-glasshouse” plants in *Saussurea* and *Rheum* L. revealed convergent evolution of this body-plan trait even within a single genus (Wang et al., 2009c; Sun et al., 2012). A recent study compared the homologous candidate genes with the same expression changes between the two “glasshouse” species in *Rheum*, *R. nobile* Hook.f. & Thoms. and *R. alexandrae* Batalin, and concluded that the “glasshouse” phenotypes may have common molecular bases underlying their convergent evolution of similar adaptive functions (Zhang et al., 2010; Liu et al., 2013). However, it is still unknown whether the convergent evolution of this phenotype has the same molecular mechanism on a larger scale and in other groups.

### BIOTIC INTERACTIONS: POLLINATOR-MEDIATED ISOLATION

Many species-rich plant lineages such as *Corydalis* Vent., *Gentiana* L., *Meconopsis*, *Pedicularis*, *Saussurea*, *Aconitum* L., and *Delphinium* L. are pollinated by bumblebees (He et al., 2006; Duan et al., 2007, 2009; Hou et al., 2008; Niu et al., 2011; Eaton et al., 2012) with congeners commonly co-occurring at local scales on the QTP. Closely related species of the plant genus *Pedicularis* co-occur geographically, share pollinators (bumblebees), and exhibit high floral diversity and species richness on the QTP. Biotic, especially pollinator-mediated, interactions among the *Pedicularis* species have been hypothesized to promote ecological sorting through recurrent selection against reproductive interference, which explains the rapid species turnover at local scales, and drives floral divergence among species of *Pedicularis* (Eaton et al., 2012). Whether pollinator-mediated reproductive interference is unique in *Pedicularis* or has played a more general role in the regional plant diversifications need to be further explored with comparative studies of pollination ecology, reproductive biology, and phylogenetic community structure. Duan et al. (2009) reported that a combination of insect and wind pollination may have played an important role in maintaining sexual reproduction of the biennial herbaceous species *Aconitum gymnandrum*, and may have allowed the species to persist in arid habitats on the QTP during Quaternary glacial periods when there were extensive oscillations of pollinator populations. Also Hou et al. (2008) compared sympatric gentian species sharing the same pollinator, yet they differ in phenology, with incomplete pollination isolation. The role of such isolation mechanisms needs to be explored in large groups, such as *Pedicularis*, *Rhododendron*, and *Primula* L.

### POLYPLOIDY

Polyploidy is considered to be one of the most important cytogenetic mechanisms in plant evolution (Stebbins, 1940, 1971; Mayrose et al., 2011). Polyploidy is relatively common in plants of cold climates with harsh environments, especially in alpine and arctic regions (Löve and Löve, 1967; Grant, 1981; Stebbins, 1984; Brochmann et al., 2004). A common explanation of this pattern is that polyploids possess broader ecological tolerances than their diploid parents/relatives and may be more efficient to adapt to new habitats (Stebbins, 1984; Soltis and Soltis, 1999, 2000; Brochmann et al., 2004; Otto, 2007). The QTP has the highest mountains and the most extreme environment in the world, and the advancement and retreat of ice sheets and glaciers since the Quaternary have created new habitats for vegetative colonization of polyploids.

No statistic chromosomal data are available from the QTP, however, Nie et al. (2005a) have indicated that polyploidy may have played only a minor role in the evolutionary diversification in the Hengduan Mountains, which is located on the eastern border of the QTP. Wu et al. (1995) also reported their observations on the low frequencies of polyploids in the alpine flora of the QTP. Based on the statistical analysis of the chromosome data of 552 taxa of native angiosperms, Nie et al. (2005a) reported that the frequency of infrageneric polyploidy is only 22%. Several highly diversified groups, such as *Cremanthodium* (Liu et al., 2001), *Ligularia* (Liu et al., 2006), and *Delphinium* (Yuan and Yang, 2008), also show a low proportion of polyploidy on the QTP and adjacent areas. Examples from other endemic genera (e.g., *Syncalathium*, *Soroseris*, *Solms-laubachia* Muschl., *Tibetia* (Ali) H.P.Tsui, and *Nomocharis* Franch.) again indicated that polyploidy, especially infrageneric polyploidy, may have played a minor role on the evolutionary diversification of these plants in this region (Xie et al., 1992; Nie et al., 2002; Yue et al., 2004; Zhang et al., 2007, 2009a).

However, cytological investigations on several other groups have suggested the importance of polyploidy. *Leontopodium* is the second largest genus within the Asian Gnaphalieae (Asteraceae) and is most speciose on the QTP and its adjacent areas in SW China. The chromosome numbers and karyomorphology of five species from this region suggests that the basic chromosome number of *Leontopodium* is *x* = 14, followed with dysploidy numbers (e.g., *x* = 12, 13), and that three of the five species examined are polyploids (Meng et al., 2012). Dysploidy and polyploidy observed in *Leontopodium* may have facilitated the adaptation of *Leontopodium* to diverse habitats on the QTP. Luo et al. (2011) examined the chromosomes of *Silene* L. from high elevations of the Hengduan Mountains, and reported the basic chromosome number as *x* = 12. So far only seven species of *Silene*, which has its diversity center in the Hengduan Mountains, have been reported with cytological data, of which three species are polyploids. Chen et al. (2007) reported 10 of the 18 *Buddleja* L. species in the Hengduan Mountains to be polyploids. Several other cases also found that polyploidy is common in plants in the alpine areas of the eastern Himalaya and the Hengduan Mountains, such as in *Aconitum* subgenus *Lycoctonum* (DC.) Peterm. (Yuan and Yang, 2006), *Rheum* (Liu et al., 2010), and *Anaphalis* (Meng et al., 2010). Wu et al. (2010) suggested that the tetraploid cytotypes of *Allium* *przewalskianum* Regel had evolutionary advantages over diploids in colonizing and/or surviving the arid habitats of the QTP.

Clearly polyploidy has played a role in the evolutionary diversification of some plant groups on the QTP. Further investigations of characteristic groups of the QTP region, possibly with different evolutionary histories, are needed to more precisely evaluate the role of polyploidy in the speciation on the QTP, in comparison to other alpine regions.

## BIOGEOGRAPHIC CONNECTIONS OF THE QTP WITH OTHER REGIONS

**FIGURE 2:**
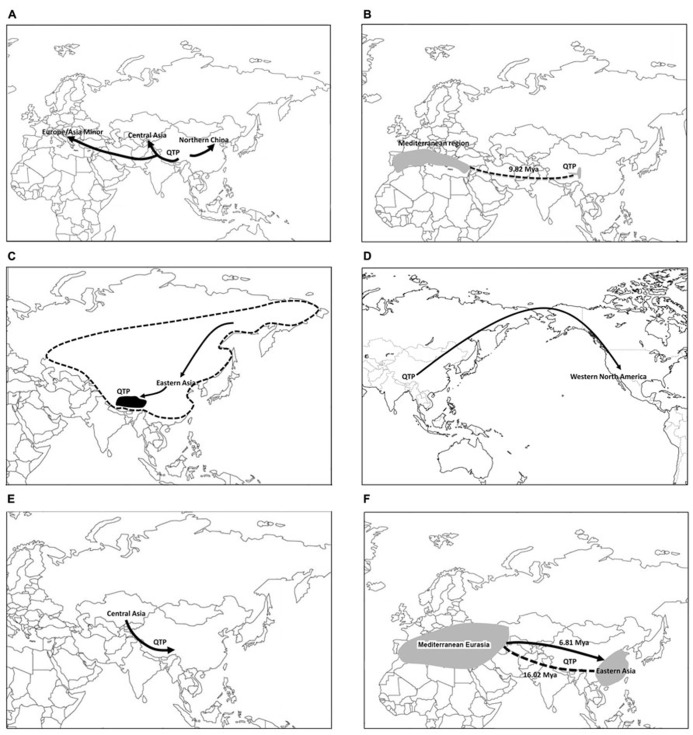
**Major patterns of biogeographic diversification of plants on the Qinghai-Tibetan Plateau: (A)** the QTP as a biogeographic source area in Eurasia with examples of *Gentiana* sect. *Cruciata* (Wang et al., 2004) and *Hippophae rhamnoides* (Jia et al., 2012); **(B)** close biogeographic connections of the QTP with Mediterranean Eurasia, as exemplified by *Mandragora* (Tu et al., 2010); **(C)** Hengduan–Himalayan forest regions of the QTP showing close biogeographic relationships with Arcto-Tertiary floristic elements in eastern Asia, as exemplified by the radiation of *Maianthemum* on the QTP following the migration from the Sino-Japanese floristic region (Meng et al., 2008); **(D)** the QTP and western Cordillera forest region of western North American, as shown in *Kelloggia* (Nie et al., 2005b); **(E)** migration of the QTP plants from Central Asia, as inferred from *Solms-laubachia* (Yue et al., 2009); and **(F)** the QTP as a major biogeographic barrier for plant diversification in Eurasia, as shown by examples of two Mediterranean–eastern Asian disjunctions in *Pistacia* (Yi et al., 2008; Xie et al., 2014).

The Tethyan Tertiary flora and the Arcto-Tertiary flora have been suggested as important sources of the QTP alpine and forest floras (Sun, 2002a,b; Zhou et al., 2013b). Examples of Tethyan Tertiary elements include *Helleborus* L. (Sun et al., 2001), *Incarvillea* Juss. (Chen et al., 2005), *Mandragora* L. (Tu et al., 2010), *Hyoscyamus* L. (Tu et al., 2010), *Pistacia* L. (Yi et al., 2008; Xie et al., 2014), and *Ruta *L. (Salvo et al., 2010). The Arcto-Tertiary elements are exemplified by *Rhododendron* L. (Kron, 1997), *Aralia* L. (Wen et al., 1998; Wen, 2011), *Panax* L. (Wen and Zimmer, 1996; Lee and Wen, 2004); *Circaea* L. (Xie et al., 2009), *Triosteum* L. (Gould and Donoghue, 2000), *Maianthemum* Wiggers (Meng et al., 2008), and *Astilbe* Buch.-Ham. ex D.Don (Zhu et al., 2013). The Turgai Strait stretching from the Arctic Ocean to the Tethys Seaway separated the European and Asian floras through the Paleocene to the early Oligocene (Tiffney, 1985). The closure of the Turgai Strait brought about a drier and cooler continental climate across Central Asia, and allowed extensive biogeographic exchanges between Asia and Europe (Tiffney and Manchester, 2001). So far a relatively few number of studies have been conducted to explore the biogeographic significance of the QTP in the Northern Hemisphere based on modern phylogenetic evidence. We thus attempt a review to summarize the available evidence (also see **Figures 2A–F**) and hope that this will present the QTP as an exciting region for colleagues beyond eastern Asia.

### THE QTP AS A BIOGEOGRAPHIC SOURCE AREA IN EURASIA (Figure 2A)

Several lineages have been inferred to have originated on the QTP and subsequently migrated into the other regions of Eurasia, i.e., the out-of-QTP hypothesis (e.g., in Wang et al., 2004; Fan et al., 2013b; Zhang et al., 2013). One case is *Gentiana* sect. *Cruciata* Gaudin, which is mainly distributed in alpine areas in Eurasia, with the greatest species diversity on the QTP (Zhang et al., 2009b). The phylogenetic analyses support the monophyly of the section with five major clades identified. The basalmost clade comprised three species that were endemic to the QTP. The clade that diverged second was represented by three Central Asian species. The European clade, containing only *G. cruciata* L., grouped with the remaining two clades constituting species from the QTP and Central Asia. The biogeographic analyses and divergence time estimates suggested that this section diversified initially on the QTP within 4 Ma. The results support the biogeographic significance of the QTP for alpine plant evolution, and the lineage then migrated to Central Asia and Europe from the QTP and/or western China after the Pliocene when global temperatures decreased.

*Lagotis* J.Gaertn. is a small genus of herbaceous plants of Plantaginaceae containing about 28 species mainly occurring on the QTP, Central Asia, arctic and subarctic Asia, and Northwest America. The centers of diversity of this genus are on the QTP (15 endemic species) and in Central Asian Mountains (eight endemic species). Molecular biogeographic analyses suggested the origin of *Lagotis* on the QTP in the Miocene with its subsequent radiations from the Miocene to Pleistocene. The diversification of *Lagotis *probably took place predominantly in the QTP and it then spread to the Central Asian highlands, supporting the “Central Asiatic Highland Corridor” as proposed by Ohba (1988), followed by the northward migration into the arctic (Li et al., 2014).

A similar pattern has been documented in *Rhodiola* L. (Crassulaceae; Zhang et al., 2013), *Androsace* L. (Primulaceae; Wang et al., 2004), *Leontopodium* (Blöch et al., 2010; Safer et al., 2011), *Anaphalis* (Nie et al., 2013), and *Koenigia* L. (Fan et al., 2013b).

The out-of-QTP scenario was also shown at the shallower scale. For example, haplotype distribution pattern, as well as coalescence and molecular variance analyses of the fern *Lepisorus clathratus* Ching from mainly the QTP and north-central China suggest that populations in the Hengduan Mountains possess the highest genetic diversity, whereas a single haplogroup is found across the north-central region of China, supporting the hypothesis of recolonization from the glacial refugia of the QTP into the north-central regions of China northward into the Altai during interglacial periods (Wang et al., 2011b). Other cases of the out-of-QTP colonization pattern within a species have been reported, such as in *Hippophae rhamnoides* L. (Jia et al., 2012) and *Sophora davidii* (Fan et al., 2013a).

### CLOSE BIOGEOGRAPHIC CONNECTIONS OF THE QTP WITH THE MEDITERRANEAN EURASIA (Figure 2B), AND/OR NORTH AMERICA

The Mediterranean regions of Central and West Asia have been suggested to be an important biogeographic source region for plant lineages on the QTP (Sun, 2002a). An excellent example of this disjunct pattern is shown by the genus *Mandragora* (five species, Solanaceae). Tu et al. (2010) resolved the genus into two clades: one including *M. officinarum* L., *M. autumnalis* Bertol., and *M. turcomanica* Mizgir. from the Mediterranean region and the other clade consisting of *M. caulescens* C.B. Clarke and *M. chinghaiensis* K.Z. Kuang & A.M. Lu from the QTP region (**Figure 2B**). The origin of this disjunct pattern was interpreted as a result of the interruption of a once more continuous distribution before the uplift of the QTP (Sun, 2002a). The uplift of the QTP was mainly in the Miocene (Harrison et al., 1992) and is the major late Tertiary geologic event in Eurasia. The age of the disjunction in the mandrakes was estimated to be 9.82 Ma [95% highest posterior density (HPD): 4.40–16.18 Ma], consistent with a Miocene vicariance hypothesis (Tu et al., 2010).

Sun et al. (2001) explored the biogeographic relationships of *Helleborus* L. (ca. 16 species, Ranunculaceae). They found that species of the large section *Helleborastrum* Spach mostly from southern Europe and the Mediterranean region formed a clade with the monotypic section *Dicarpon* Ulbrich consisting of *H. thibetanus* Franch. from the QTP. Using a general molecular clock, the divergence of this disjunction was dated to be 22.96 Ma in the early Miocene. The study provides support of the vicariance hypothesis of an earlier more continuous Tethyan distribution of *Helleborus* across Eurasia (Wu, 1988). Because the dispersal agents of *Helleborus* seeds are normally ants, a long distance-dispersal scenario was considered unlikely.

The genus *Coriaria* Niss. ex L. (Coriariaceae) is morphologically highly variable with 5–20 species recognized by various authors (see Good, 1930; Yokoyama et al., 2000). The single Mediterranean species *C. myrtifolia* L. forms a clade with the Himalayan *C. nepalensis* Wall. and *C. terminalis* Hemsl. on the QTP. This QTP–Mediterranean disjunct clade is then sister to *C. japonica* A. Gray–*C. intermedia* Matsum. clade of eastern to SE Asia. Yokoyama et al. (2000) suggested the migration of *Coriaria* in the direction from Asia to the Mediterranean region and the climatic changes associated with the glaciation and drying in the Cenozoic as the explanation of this Mediterranean–Asian disjunction.

Subgenus *Amerallium* Traub of the large genus *Allium* L. (Amaryllidaceae) shows an early vicariance between the QTP and the Mediterranean region (Li et al., 2010a). This Eurasian clade is then sister to the clade of the North American *Amerallium* taxa. Eastern Asia was inferred to be the most likely ancestral area of the *Amerallium* clade.

*Ephedra* L. has been suggested to have originated in the Mediterranean Eurasia (MEA; Ickert-Bond et al., 2009). It then diversified in Asia and the New World with two distinct lineages on the QTP (the southern QTP lineage and the eastern QTP lineage) dated to the middle or late Miocene (Qin et al., 2013; also see Ickert-Bond et al., 2009).

*Juniperus* L. is a coniferous genus with 67 recognized species and is major component of arid and semi-arid tree/shrub ecosystems in the Northern Hemisphere (Adams, 2008). The genus is monophyletic and is classified into three monophyletic sections: sect. *Caryocedrus* Endl. (one species in the Mediterranean), sect. *Juniperus* (nine species in eastern Asia and the Mediterranean plus one circumboreal species *J. communis* L., and sect. *Sabina* Spach (56 species distributed in southwestern North America, Asia, and the Mediterranean region, extending to Africa and the Canary Islands). The most comprehensive phylogenetic and biogeographic study of *Juniperus* was by Mao et al. (2010), who sampled 51 of the 67 recognized species and employed nine cpDNA markers. Most QTP species of *Juniperus* belong to sect. *Sabina*. Mao et al. (2010) showed that sect. *Sabina* has five clades. Clade I contains *Juniperus pseudosabina* Fisch. & C.A.Mey. from Central Asia (Xinjiang, China) plus all Himalayan/QTP alpine species except *J. microsperma* (W.C.Cheng & L.K.Fu) R.P.Adams (in clade III) and *J. gaussenii* Cheng. Clade II comprises the serrate-leaved junipers of North America and is sister to clade I. Clade III includes the smooth-leaved American species, the Eurasian *J. sabina* L., the middle Asian *J. semiglobosa* Regel, and the QTP endemic *J. microsperma*. Clade IV comprised the *J. chinensis* L. complex (including the QTP species *J. gaussenii*) from eastern Asia, *J. thurifera* L. from Europe, *J. excelsa* Pursh from the eastern Mediterranean, *J. polycarpos* K.Koch (from west Himalaya to Caucasus) and *J. procera* Hochst. ex Endl. (eastern Africa and southern Arabia). Clade V contained only the Mediterranean *J. phoenicea *Pall. The ancestral area of *Juniperus* is in Eurasia, but could be Europe, Asia, or a combination of these two. The phylogeny of *Juniperus* clearly indicates the close biogeographic relationship of the QTP with Central Asia, as well as with North America in this semi-desert xeric group and supports the Madrean–Tethyan biogeographic connection (Wen and Ickert-Bond, 2009).

### HENGDUAN–HIMALAYAN FOREST REGIONS OF THE QTP SHOWING CLOSE BIOGEOGRAPHIC RELATIONSHIPS WITH ARCTO-TERTIARY FLORISTIC ELEMENTS IN EASTERN ASIA AND EASTERN NORTH AMERICA (Figure 2C)

Recent phylogenetic evidence from various plant lineages supports the view that the Hengduan–Himalayan forest regions of the QTP contain many floristic elements originated from the Arcto-Tertiary flora as well as preserved some unique relict taxa (Wen et al., 1996; Wen, 1999; Sun, 2002b; Xiang et al., 2004). The Arcto-Tertiary floristic elements have been well preserved in eastern Asia (especially in the Sino-Japanese floristic region) as well as eastern North America (Wen, 1998, 1999, 2001; Wen et al., 2010; Harris et al., 2013). Below we briefly discuss a few examples of the QTP plants showing the Arcto-Tertiary floristic affinities. Analyses of large genera such as *Rhododendron* with adequate sampling from the QTP are still lacking. Some groups, e.g., *Angelica* L., show a particularly complex biogeographic history (Liao et al., 2012; also see Feng et al., 2009).

*Maianthemum* (Asparagaceae) consists of ca. 35 species widely distributed in the Northern Hemisphere. The Hengduan–Himalayan regions represent a major center of diversity for the genus with 13 species. Most *Maianthemum* species from the Hengduan–Himalayan regions form a well-supported clade nested within several basal grades including species from northeastern Asia and the New World (Meng et al., 2008). The phylogenetic and biogeographic analyses suggest a most likely New World origin and a north-to-south migration within eastern Asia. Species of *Maianthemum* radiated in the Hengduan–Himalayan regions of the QTP within the last 2 Ma, as estimated by Meng et al. (2008), supporting the QTP as a speciation pump for such an Arcto-Tertiary group. The QTP lineage shows a close affinity with the Sino-Japanese elements of the genus, especially those from Central to SW China.

*Panax* L. (the ginseng genus, Araliaceae) consists of about 15–18 species with two from eastern North America and the remaining from eastern Asia spanning from Japan to the Hengduan Mountains and the eastern Himalaya. Molecular phylogenetic results (Lee and Wen, 2004; Zuo et al., 2011) showed a large clade of *Panax* taxa from the Hengduan–Himalayan region as well as the warm temperate to subtropical areas of the Sino-Japanese region (the *P. bippinatifidus* Seem. superspecies complex). In *Panax*, the Hengduan–Himalayan region is found to be an active diversification center with recently differentiating taxa, as well as a refugium for some unique and early diverged species (e.g., *P. pseudoginseng* Wall.). A similar pattern between the taxa from the Hengduan–Himalayan region and the Sino-Japanese region has been recorded in *Aralia* L. sect. *Aralia* (Wen et al., 1998; Wen, 2011), with recently diversified species (e.g., *A. apioides* Hand.-Mazz. and *A. atropurpurea* Franch.) in the Hengduan Mountains and some early diverged taxa (e.g., *A. cachemirica* Decne. and *A. tibetana* G. Hoo) in the Himalayan Mountains.

*Astilbe* (Saxifragaceae) consists of about 20 species with the center of diversity clearly in the Sino-Japanese floristic region with only two species found in the Himalayan–Hengduan regions of the QTP, and one species in eastern North America (Pan, 1995). The phylogenetic and biogeographic analyses of the genus (Kim et al., 2009; Zhu et al., 2013) support three major clades, a clade centered in the northern part of the Sino-Japanese region, a clade centered in the central and southern part of the Sino-Japanese region, and a clade of *A. rivularis* Buch.-Ham. ex D.Don primarily distributed in the Himalayan–Hengduan regions of the QTP extending to SE Asia. The early divergence of *A. rivularis* in the mid-Miocene and the relatively basal position of this widespread species suggest that the Himalayan–Hengduan regions of the QTP served as a refugium for *Astilbe* evolution in the mid Tertiary. The QTP *A. rivularis* is also suggested to be sister to the eastern North American relict species *A. biternata* (Vent.) Britton. The other QTP species *A. rubra* Hook.f. & Thoms. dispersed into southern QTP in the late Miocene or early Pliocene from the Sino-Japanese region.

*Triosteum* L. (Caprifoliaceae) is a small genus of six species with three in eastern Asia and three in eastern North America (Gould and Donoghue, 2000). *Triosteum himalayanum* Wall. of the QTP was found to be sister to *T. pinnatifidum* Maxim. from the Sino-Japanese region. The three North American species formed a clade sister to the *T. himalayanum*–*T. pinnatifidum* clade. *Triosteum sinuatum* Maxim. from Central Japan and NE China was the first diverged species of the genus. The QTP species *Triosteum himalayanum* thus occupies a relatively advanced position within *Triosteum*.

The genus *Podophyllum* L. (Berberidaceae) consists of two intercontinental disjunct species: *P. peltatum* L. from eastern North America and *P. hexandrum* Royle from the Himalayan–Hengduan Mountains (Liu et al., 2002b), with the latter species sometimes recognized as its own genus *Sinopodophyllum* T.S. Ying (Li et al., 2011). The monophyly of this disjunct genus of two species has been supported, and *Podophyllum* is then sister to the genus *Dysosma* Woodson native to the Sino-Japanese region to South China and neighboring area of North Vietnam (Wang et al., 2007a). The relationships in the *Podophyllum*–*Dysosma*–*Diphylleia* clade show a close biogeographic relationship among the forest regions of the Himalayan–Hengduan Mountains, the Sino-Japanese floristic region, and eastern North America. The wide disjunction in *Podophyllum* likely represents a relict distribution dating back to the late Miocene (Wang et al., 2007a).

Even though the eastern Asian–North American biogeographic disjunctions have attracted many research interests in the last 15 years (see review by Wen et al., 2010), the QTP taxa have often not been incorporated in the biogeographic analyses of this classical biogeographic pattern, or the discussions often did not elaborate on the biogeographic assembly within eastern Asia or Asia overall. Future studies clearly need to place the QTP taxa into the perspectives of Arcto-Tertiary plant biogeography.

### THE QTP AND WESTERN CORDILLERA FOREST REGION OF WESTERN NORTH AMERICA (Figure 2D)

The alpine flora of the QTP occasionally shows biogeographic affinities with the forest elements of the western Cordillera of North America. The genus *Kelloggia* Torr. ex Hook.f. (Rubiaceae) has two species: *K. chinensis* Franch. from the Hengduan Mountains and *K. galioides* Torrey from the western United States. Ecologically *K. chinensis* occurs in alpine meadows or open places or shrublands along the stream of high mountains (about 3000 masl), whereas *K. galioides* grows in open places in coniferous forests (1100–3000 masl), sometimes among rocks or along streams. The biogeographic and dating analyses by Nie et al. (2005b) suggest that the current intercontinental disjunct distribution of *Kelloggia* may be best explained to be via a long-distance dispersal from Asia into western North America after the uplift of the Himalayan Mountains and the western Cordillera of North America in the late Miocene.

The QTP harbors high diversity of conifers (Ran et al., 2006). The most extensively analyzed conifer group is the spruce genus *Picea* A.Dietr. (ca. 35 species), which often occupy forest habitats (also see Meng et al., 2007; Li et al., 2010b, 2013a; Du et al., 2011; Zou et al., 2012). The regions of highest *Picea* diversity are the western Cordillera of North America and the QTP. The most recent phylogenetic analyses of the genus using mitochondrial, plastid, and nuclear data (Lockwood et al., 2013) suggest that the 9 or 10 *Picea* species of the QTP region formed a major clade, with one western Cordillera species in North America (*P. breweriana* S.Watson) and one narrowly distributed species from Taiwan (*P. morrisonicola* Hayata). The QTP clade of the 9–10 species was estimated to have diverged around 19.8 Ma. The ancestral area of *Picea* has been highly debated (Ran et al., 2006; Bouillé et al., 2011; Lockwood et al., 2013). If we accept the Asian origin hypothesis (Wright, 1955; Lockwood et al., 2013), the diversification of the large QTP clade may be interpreted as having a QTP origin with one dispersal into western North America and another dispersal into Taiwan in the early Miocene. Nevertheless, the phylogenetic position of the western North American *Picea breweriana* has been controversial and it may represent a relict lineage (Lockwood et al., 2013).

### MIGRATIONS/DISPERSALS FROM CENTRAL ASIA INTO THE QTP (Figure 2E)

Phylogenetic evidence supports the Central Asian origin with subsequent diversification on the QTP in several groups. This pattern is still poorly known and needs to be further explored. The extensive molecular data of *Solms-laubachia* Muschl. (Brassicaceae) suggest that the Hengduan Mountain endemics were embedded in a paraphyletic grade of species from Central Asia and the western Himalayas (Yue et al., 2009). The genus was hypothesized to have originated in Central Asia in the Pliocene, and subsequently migrated eastward into the Hengduan Mountains, the eastern border of the QTP. This relatively young group colonized sky-island, alpine scree-slope habitats that may have provided novel ecological opportunity and accelerated speciation, becoming quickly established in the Hengduan Mountains, the present center of diversity for the genus.

*Incarvillea* Juss. (Bignoniaceae) is composed of 16 species classified into four subgenera with the Himalayan and Hengduan Mountains of the QTP as the current center of diversity and a few species in Central Asia and Mongolia (Grierson, 1961). The earliest diverged taxa are endemic to Central Asia, and the most derived group, subgenus *Pteroscleris* Baillon, is distributed in the eastern Himalaya and the Hengduan Mountains (Chen et al., 2005). Chen et al. (2005) suggested the dispersal of *Incarvillea* from Central Asia to the QTP prior to the Miocene, as well as a recent radiation on the QTP, perhaps related to the uplift of the Himalayan–Hengduan massifs.

*Myricaria* Desv. is a small genus of eleven species, mostly distributed on the QTP and adjacent regions. Wang et al. (2009b) suggested a Himalayan origin of the genus. Recently Chen (2013) re-evaluated the ancestral distribution of the genus, and suggested eastern Asia (the QTP) as the ancestral origin of the genus, and the “out of eastern Asia” episodes only happened after drastic uplifts of the QTP at 5 Ma; yet based on their results, the most basally diverged species *Myricaria elegans* Royle, *M. prostrata* Hook.f. & Thomson ex Benth. & Hook.f.**and *M. pulcherrima* Batalin, as well as the outgroup *Tamarix* are mainly distributed in the western Himalaya or Xinjiang (NW China) and adjacent regions such as Kashmir and West Pakistan (Wang et al., 2006), where the flora is part of that of Central Asia, rather than eastern Asia (the QTP). The other clades on the phylogenetic tree represented younger evolutionary derivatives mostly on or outside the QTP. *Myricaria* is thus argued here to have originated from Central Asia, and it then migrated into the QTP and the adjacent regions following its origin.

### THE QTP AS A MAJOR BIOGEOGRAPHIC BARRIER FOR PLANT DIVERSIFICATION IN EURASIA

The eastern Asian-Mediterranean Tethyan biogeographic disjunction (Thompson, 1999; Sun and Li, 2003; Tu et al., 2010; Wan et al., 2013) was largely attributed to be extinctions in the Asian interior during the Miocene (Sun, 2002a; Qiao et al., 2007). The Pliocene climatic changes have been suggested to be a more plausible explanation for more recent disjunctions between Europe and Asia (Fiz-Palacios et al., 2010; Tu et al., 2010; Carlson et al., 2012). The genus *Pistacia* represents an example of a widespread ancestral area of the Old World clade of *Pistacia* in MEA and eastern Asia (Yi et al., 2008; Xie et al., 2014). Two independent eastern Asian–MEA disjunction events were inferred within the clade of Old World species. The divergence between the eastern Asian *P. weinmannifolia* J.Poiss. ex Franch.–*P. cucphuongensis* T.D.Dai clade and the clade of the remaining Old World species was estimated to be 16.02 mya (95% HPD: 10.90–23.41 mya) at the boundary of Early and Middle Miocene. The timing of this divergence was consistent with the recent major uplifts of the QTP between the early Miocene to the Pleistocene (Harrison et al., 1992; Guo et al., 2002; Spicer et al., 2003). The second disjunction was between *Pistacia chinensis* Bunge and the *P. integerrima* J.L.Stewart–*P. palaestina* Boiss.–*P. terebinthus* Mill. clade of MEA. *Pistacia chinensis* from eastern Asia is nested within the clade of MEA species. One dispersal event from MEA to eastern Asia was inferred to account for the distribution of *P. chinensis*. This dispersal event was suggested to have occurred during the late Miocene (6.81 mya, 95% HPD: 3.59–10.20 mya). Migration between Europe and Asia has been hypothesized to be primarily in an east–west direction (Tiffney and Manchester, 2001), which has been supported by molecular evidence in *Rhus* L. (Yi et al., 2004). Yet *Scabiosa* L. was inferred to have migrated from west to east (Carlson et al., 2012). The biogeographic diversification of *Pistacia* *chinensis* and its close relatives represents another example of such a west-to-east migration direction across Eurasia.

*Scabiosa* (Dipsacaceae) exhibits an unusual Old World disjunction in Asia (12 species), Europe (primarily in the Mediterranean Basin, ca. 14 taxa), and eastern and southern Africa (ca. eight species).* Scabiosa* was inferred to have originated in the middle to late Miocene in Mediterranean Europe, followed by independent migrations into Asia and Africa (Carlson et al., 2012). The split between Europe and Asia in *Scabiosa* was inferred to be 2.3–6.6 Ma. Even though Carlson et al. (2012) did not rule out the vicariance hypothesis, the Pliocene climate fluctuations were proposed to be a more likely explanation for this disjunction, as is consistent with studies on other plant groups exhibiting more recent disjunctions between Europe and Asia (see Fiz-Palacios et al., 2010; Tu et al., 2010).

The high mountains in the QTP and adjacent areas apparently have served as biogeographic barriers for some plant groups. For example, a clade including *Scapiarabis* M.Koch, R.Karl, D.A.German & Al-Shehbaz, *Botschantzevia* Nabiev, *Arcyosperma* O.E.Schulz, *Parryodes* Jafri, and *Borodiniopsis* D.A.German, M.Koch, R.Karl & Al-Shehbaz of tribe Arabeae of Brassicaceae forms a circle around the Asian high mountain system, which is interrupted and separated by genetically distinct members of the sister clade that includes *Baimashania* Al-Shehbaz, *Pseudodraba* Al-Shehbaz, D.A.German & M.Koch, and *Sinoarabis* R.Karl, D.A.German, M.Koch & Al-Shehbaz (Koch et al., 2012). The unique biogeographic circle predates the Pleistocene with these genera dated to be of Pliocene origin.

### THE QTP AND THE SOUTHERN HEMISPHERE

Most taxa endemic to the QTP have been hypothesized to have originated *in situ* or from areas in the Northern Hemisphere, as discussed above. *Nannoglottis* Maxim. (Asteraceae), with ca. eight species endemic to the QTP, is however found to be monophyletic and supported as an isolated genus as the first diverged lineage of the tribe Astereae, which has a Southern Hemisphere origin (Liu et al., 2002a; Brouillet et al., 2009; V. A. Funk, personal communication). Long-distance dispersal using SE Asia as a steppingstone from Southern Hemisphere to the QTP was suggested the most likely explanation for this unusual biogeographic link of *Nannoglottis* with the Southern Hemisphere (Liu et al., 2002a). The molecular phylogeny of the genus further identifies two distinct clades, the “alpine shrub” and the “coniferous forest” clades, with their divergence estimated at ca. 3.4 Ma when the plateau began its first large-scale uplift and the coniferous vegetation began to appear. Most species of the “coniferous forest” clade of the genus are estimated to have originated from 1.02 to 1.94 Ma, when the second and third uplifts of the QTP occurred, the climate oscillated and the habitats were changed drastically. The genus *Koenigia* has most species on the QTP and the adjacent areas; yet one species *K. islandica* was widespread in the arctic and alpine areas of the Northern Hemisphere as well as southern South America (Hedberg, 1997). This South American distribution of *K. islandica* is apparently due to a recent long-distance dispersal from the Northern Hemisphere, most likely not directly from the QTP (Fan et al., 2013b). Biogeographic connections between the QTP and the Southern Hemisphere are rare and need to be explored in future studies.

## PROSPECTUS

Allopatric differentiations (vicariance) have been supported to be important in the plant diversification on the QTP. Species radiations on the QTP can provide unique insights into speciation, species interactions and diversifications, and morphological innovations, due to the relative recent geologic history of the QTP and its extraordinarily rich biodiversity. Little is still known on the patterns associated with the evolutionary radiations through integrative studies of phylogeny, morphological innovations, geography, diversification rates, ecology, and cytology of the QTP plants. Also insights into the patterns and processes of species diversifications have been hampered by the often poorly resolved species phylogenies. Multilocus approaches using many genes via conventional Sanger sequencing should remain as a useful approach for the near future. Nevertheless, the next-generation sequencing (NGS) approaches now have shown great potential for efficiently sampling entire genomes of plant species for phylogenetically informative variation. NGS approaches such as restriction-site associated DNA sequencing (RADseq; Miller et al., 2007; Baird et al., 2008; Emerson et al., 2010; Peterson et al., 2012), or genotyping-by-sequencing (GBS; Elshire et al., 2011) allow the regions adjacent to restriction sites to be surveyed with deep coverage. The RADseq data have been recently utilized to explore the phylogenetic relationships of recently diverged taxa or radiations (Eaton and Ree, 2013; Wagner et al., 2013) and they can be employed to study the species diversifications on the QTP. Sequences of whole plastomes or a large number of rapidly evolving regions of plastomes have been obtained via NGS to resolve rapid radiations (e.g., Fior et al., 2013; Li et al., 2013b). Other NGS approaches such as RNA-Seq (Wang et al., 2009d) may provide insights into the developmental mechanisms of some of the fascinating morphological innovations associated with evolutionary radiations. The biogeographic relationships of the QTP with other regions of the Northern Hemisphere need to be explored more extensively to better understand the role of the QTP in the evolutionary diversification of plants (also see Lexer et al., 2013). Integrative multidimensional analyses of phylogeny, morphological innovations, geography, ecology, development, species interactions and diversifications, and geology (Wen et al., 2013) are needed and should shed insights into the patterns of evolutionary assembly and radiations in this fascinating region.

## Conflict of Interest Statement

The authors declare that the research was conducted in the absence of any commercial or financial relationships that could be construed as a potential conflict of interest.
